# Highly Clonal Structure and Abundance of One Haplotype Characterise the *Diplodia sapinea* Populations in Europe and Western Asia

**DOI:** 10.3390/jof7080634

**Published:** 2021-08-04

**Authors:** Kalev Adamson, Marili Laas, Kathrin Blumenstein, Johanna Busskamp, Gitta J. Langer, Darta Klavina, Anu Kaur, Tiit Maaten, Martin S. Mullett, Michael M. Müller, Emília Ondrušková, Allar Padari, Enn Pilt, Taavi Riit, Halvor Solheim, Liina Soonvald, Leho Tedersoo, Eeva Terhonen, Rein Drenkhan

**Affiliations:** 1 Institute of Forestry and Rural Engineering, Estonian University of Life Sciences, 51014 Tartu, Estonia; Marili.Laas@student.emu.ee (M.L.); tiit.maaten@emu.ee (T.M.); allar.padari@emu.ee (A.P.); rein.drenkhan@emu.ee (R.D.); 2 Forest Pathology Research Group, Department of Forest Botany and Tree Physiology, Faculty of Forest Sciences and Forest Ecology, Georg-August-University, 37073 Göttingen, Germany; kathrin.blumenstein@uni-goettingen.de (K.B.); terhonen@uni-goettingen.de (E.T.); 3 Section Mycology and Complex Diseases, Department of Forest Protection, Northwest German Forest Research Institute, 37079 Göttingen, Germany; johanna.busskamp@nw-fva.de (J.B.); gitta.langer@nw-fva.de (G.J.L.); 4 Latvian State Forest Research Institute Silava, Rigas 111, LV 2169 Salaspils, Latvia; darta.klavina@silava.lv; 5 Tallinn Botanic Garden, Kloostrimetsa Tee 52, 11913 Tallinn, Estonia; anu.kaur@botaanikaaed.ee; 6 Phytophthora Research Centre, Department of Forest Protection and Wildlife Management, Faculty of Forestry and Wood Technology, Mendel University in Brno, Zemědělská 3, 613 00 Brno, Czech Republic; martin.mullett@mendelu.cz; 7 Bioeconomy and Environment, Natural Resources Institute Finland (Luke), P.O. Box 2, 00791 Helsinki, Finland; micms.muller@gmail.com; 8 Department of Plant Pathology and Mycology, Institute of Forest Ecology Slovak Academy of Sciences, 949 01 Nitra, Slovakia; ondruskova@ife.sk; 9 Estonian Environment Agency, Mustamäe Tee 33, 10616 Tallinn, Estonia; Enn.Pilt@gmail.com; 10 Center of Mycology and Microbiology, Institute of Ecology and Earth Sciences, University of Tartu, 14a Ravila, 50411 Tartu, Estonia; taavi.riit@ut.ee (T.R.); leho.tedersoo@ut.ee (L.T.); 11 Norwegian Institute of Bioeconomy Research, 1431 Ås, Norway; halvor.solheim@nibio.no; 12 Institute of Agricultural and Environmental Sciences, Estonian University of Life Sciences, 51014 Tartu, Estonia; liina.soonvald@emu.ee

**Keywords:** *Sphaeropsis sapinea*, *Diplodia pinea*, *Diplodia africana*, population genetics, species-specific PCR primer, forest pathogens, multilocus haplotyping, invasive pathogen, global trade, climate change

## Abstract

*Diplodia sapinea* is a cosmopolitan endophyte and opportunistic pathogen having occurred on several conifer species in Europe for at least 200 years. In Europe, disease outbreaks have increased on several *Pinus* spp. in the last few decades. In this study, the genetic structure of the European and western Asian *D. sapinea* population were investigated using 13 microsatellite markers. In total, 425 isolates from 15 countries were analysed. A high clonal fraction and low genetic distance between most subpopulations was found. One single haplotype dominates the European population, being represented by 45.3% of all isolates and found in nearly all investigated countries. Three genetically distinct subpopulations were found: Central/North European, Italian and Georgian. The recently detected subpopulations of *D. sapinea* in northern Europe (Estonia) share several haplotypes with the German subpopulation. The northern European subpopulations (Latvia, Estonia and Finland) show relatively high genetic diversity compared to those in central Europe suggesting either that the fungus has existed in the North in an asymptomatic/endophytic mode for a long time or that it has spread recently by multiple introductions. Considerable genetic diversity was found even among isolates of a single tree as 16 isolates from a single tree resulted in lower clonal fraction index than most subpopulations in Europe, which might reflect cryptic sexual proliferation. According to currently published allelic patterns, *D. sapinea* most likely originates from North America or from some unsampled population in Asia or central America. In order to enable the detection of endophytic or latent infections of planting stock by *D. sapinea*, new species-specific PCR primers (DiSapi-F and Diplo-R) were designed. During the search for *Diplodia* isolates across the world for species specific primer development, we identified *D. africana* in California, USA, and in the Canary Islands, which are the first records of this species in North America and in Spain.

## 1. Introduction

*Diplodia sapinea* (Fr.) Fuckel [1,2] (syn. *Diplodia pinea* (Desm.) Kickx., *Sphaeropsis sapinea* (Fr.: Fr./Dyko and Sutton) is a widely distributed pathogen of conifers causing *Diplodia* tip blight. *Diplodia sapinea* causes a range of disease symptoms in conifers, including browning of needles, shoot blight, twig and branch dieback, crown wilt and bark cankers of adult trees, collar rot, root disease and damping-off of seedlings, and blue stain of sapwood in timber [3,4,5] It is an opportunistic pathogen being able to cause significant economic losses to the most susceptible tree species in nurseries, plantations and natural forests [6,7]. The pathogen’s conidia are easily vectored to new locations by wind, rain splash, insects and humans [7,8,9]. *Diplodia sapinea* is also a well-known endophyte of *Pinus* spp. shoots, buds and needles and becomes pathogenic when the host becomes stressed [10,11,12]. This ability of *D. sapinea* to exist in an asymptomatic mode hampers timing and focusing of any control measures and may increase human mediated spread to new environments. Accordingly, reliable early detection methods are needed for detection of this pathogen in planting material, allowing the prevention of further introductions into new areas. Several molecular assays such as qPCR, conventional PCR, nested conventional PCR and High-Resolution Melting Analyses (HRMA) have been developed for the detection of *D. sapinea* [13,14,15,16]. All these assays were tested on isolates from a geographic range missing, for instance East-Asia [13,14,15,16]. Thus, a new and reliable assay for molecular detection of *D. sapinea,* tested on isolates obtained from a wide geographic range, is needed.

The pathogen has a worldwide distribution [5] in temperate and subtropical conifer forests [17,18,19,20], but has only recently been detected as a pathogen in northern hemiboreal and boreal forests. *D. sapinea* was described in Sweden in 1823 as *Sphaeria sapinea* Fr. [21] but this record has not been verified using molecular tools and the first verified observation in Sweden was made just recently by Oliva et al. [22]. In Europe it has spread northward during the last two decades possibly due to global trade and climate change [22,23,24,25,26]. Annual monitoring data since 2007 suggest that *D. sapinea* was first introduced to Estonia by human activities and subsequently spread naturally all over the country [23]. It is thought that *D. sapinea* has invaded many countries via seedlings, seeds or seed lots contaminated with debris [8,27,28]. Recent climate warming may have enabled the northward spread of *D. sapinea,* as is believed to be the case for a number other pathogens which have recently been found in northern Europe, e.g., *Dothistroma septosporum, Entoleuca mammata, Hymenoscyphus fraxineus, Lecanosticta acicola, Ophiostoma novo-ulmi* [29,30,31,32,33,34].

Asexual fruiting bodies of the fungus are commonly encountered on dead host tissue [35]. The sexual state of *D. sapinea* has never been observed, but recent studies show that the pathogen has a cryptic sexual stage [24,36,37]. Sexual recombination can increase genetic diversity of a pathogen and form genotypes that are suitable to a new environment [38]. Up to now, several population studies of *D. sapinea* have been conducted, including isolates from around the world, including Australia, Argentina, Canada, Estonia, Ethiopia, France, Indonesia, Italy, Mexico, Montenegro, New Zealand, Serbia, South Africa, Spain, Sumatra, Sweden, Switzerland, Turkey and USA using RAPD markers [39,40], RFLP [41], vegetative incompatibility [42,43,44] and SSR markers [6,35,43,45,46,47,48].

The native range of *D. sapinea* is thought to be in pine forests of the Northern Hemisphere [42,44]. However, the highest genetic diversity is observed in South Africa, when compared to populations of the Northern Hemisphere [36,45,47]. Although populations of some European countries have been characterised by molecular methods [24,43,47,48], the genetic structure of *D. sapinea* in Europe (including subpopulations recently established in northern Europe) is still poorly known.

The aim of this study was to characterise the genetic diversity of *D. sapinea* in Europe in order to improve the understanding of the pathogen’s spread across the continent. According to our experience, existing species-specific DNA primers fail to identify all isolates of *D. sapinea,* and therefore, we started this research by developing a new primer-pair and testing it with an extensive set of isolates including isolates obtained from a geographically wide range. Isolates identified as *D. sapinea* using this new primer-pair were characterised using microsatellite markers and mating type determinations. Diversity was determined both at a country (including 1–11 sites each) level and within single trees. Genetic characteristics of subpopulations were compared to the present disease situation in each country. 

## 2. Materials and Methods

### 2.1. Sample Collection and Disease Severity

**Figure 1:**
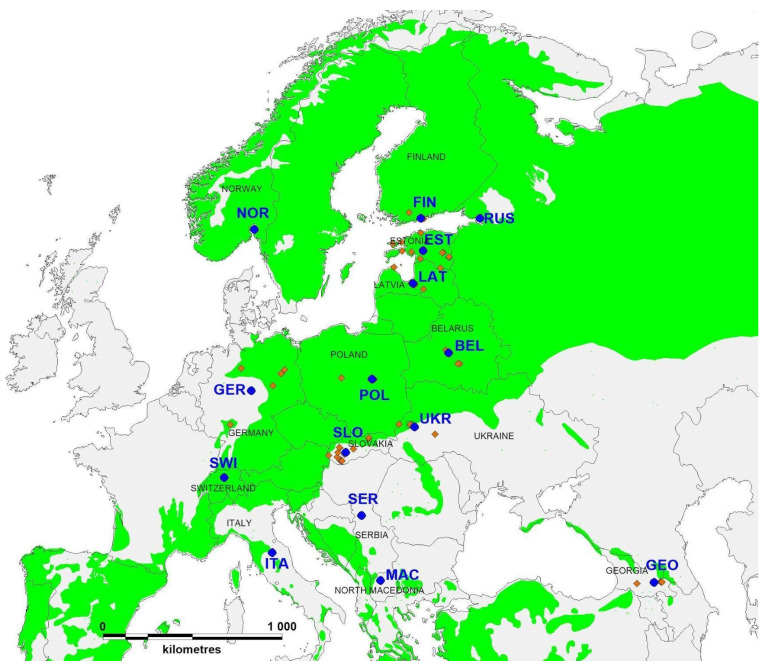
Map of sampling sites of *Diplodia sapinea*. Green shading indicates the distribution of the genus *Pinus* in Europe [49]. Red symbols indicate sampling sites (where several sites/countries were sampled); blue symbols indicate subpopulations and weighted geographical midpoints of sampling sites. Subpopulations are coded according to countries as follows: Belarus—BEL, Estonia—EST, Finland—FIN, Georgia—GEO, Germany—GER, Italy—ITA, Latvia—LAT, North Macedonia—MAC, Norway—NOR, Poland—POL, European part of Russia—RUS, Serbia—SER, Slovakia—SLO, Switzerland—SWI and Ukraine—UKR.

Pine needles, cones and shoots with and without *Diplodia sapinea* pycnidia were collected from one or several locations in each of 15 countries: Belarus, Estonia, Finland, Georgia, Germany, Italy, Latvia, North Macedonia, Norway, Poland, European part of Russia, Serbia, Slovakia, Switzerland and Ukraine (Figure 1 and Suppl. Tables S1 and S3). Sampled hosts included nine *Pinus* species, one *Pseudotsuga* species and one vector (insect) species (*Pityogenes quadridens*) (Suppl. Table S1). Samples were collected from 2011 to 2020.

Sampled cones, needles and shoots were collected from forests and urban greeneries (Suppl. Tables S1 and S3). Five isolates of *D. sapinea* were obtained from the exoskeleton of the bark beetle *Pityogenes quadridens* collected in a Norwegian *Pinus sylvestris* forest. To study the population structure on a European scale, only one fungal isolate per sampled tree was used in the analyses. To determine the genetic diversity of the pathogen at a small spatial scale, several isolates were obtained from single trees and small groups of nearby trees in Estonia and Slovakia. Additionally, several isolates per tree was used for analyses of first arrival haplotypes into Estonia.

One batch of isolates was obtained from a single *P. nigra* tree in Järvselja, south-east Estonia, from which the first *D. sapinea* record was documented in the Baltic states in 2007 [25]. In total, 16 isolates originate from this tree; five of them were isolated in 2012, three in 2013 and eight in 2018. Another batch of 14 isolates was obtained in 2012 from a small (0.7 ha) *P. nigra* stand in Muhu island, western Estonia. The third batch (10 isolates) was obtained in 2012 from six *P. sylvestris* trees on Vormsi island (on the west coast of Estonia) in a private garden, from where *D. sapinea* was found for the first time in the Baltic states on a native host, *P. sylvestris* [23]. The Estonian isolates were divided into two groups according to sampling time: (a) 2011–2012 as first arrivals of the pathogen; (b) 2013–2018 as the second wave of arrivals of the pathogen [23,25].

In Slovakia, 23 trees from 10 locations were sampled. Two to four isolates were obtained from each tree, yielding a total of 62 isolates. All the isolates were obtained in 2019 from cones of *P. nigra* or *P. sylvestris* trees. These and additional sampling sites are detailed in Suppl. Table S1.

Disease severity on native pine species during sampling was assessed in every country. Disease severity was indexed as follows: 1 = endophytic presence only, i.e., no disease outbreaks, 2 = weak local outbreaks, 3 = moderate local outbreaks (lethal for single mature trees) (Suppl. Text S1).

### 2.2. Fungal Isolations, DNA Extraction and Isolate Identification

Fungal isolations were performed according to the protocols of Mullett and Barnes [50]. Approximately 0.04 g of mycelium from the colony edge was transferred into 2.0 mL micro-centrifuge tubes and stored at −20 °C for DNA extraction. The DNA of the German samples was extracted according to Keriö et al. [51] with some modifications. Mycelium was homogenised with a Retsch MM400 homogeniser (Retsch GmbH, Haan, Germany) using metal beads (⌀ 2.5 mm). DNA was extracted using the GeneJET Genomic DNA Purification Kit (Thermo Scientific, Vilnius, Lithuania).

For the detection of *D. sapinea*, species-specific conventional PCR was performed with primers DiSapi-F and Diplo-R targeting mtSSU DNA (developed in this study, see Section 2.4). PCR reactions were carried out in 20 µL volumes: 1 µL DNA template, a final concentration of 0.4 µM of each forward and reverse primer, 4 µL 5× HOT FIREPol Blend Master Mix Ready to Load with 10 mM MgCl_2_ (Solis BioDyne, Tartu, Estonia). Cycling conditions were as follows: initial activation at 95 °C for 15 min, followed by 35 cycles of denaturation at 95 °C for 30 s, annealing at 61 °C for 30 s and elongation at 72 °C for 1 min and the final elongation at 72 °C for 10 min. All PCRs were carried out on a TProfessional Thermocycler (Biometra, Göttingen, Germany). PCR products were visualised on a 1% agarose gel (SeaKem^®^ LE Agarose, Lonza, Rockland, ME, USA) under UV light using a Quantum ST4-system (VilberLourmat SAS, Marne-la-Vallée, France).

### 2.3. ITS Sequencing

The identity of *Diplodia sapinea*, *D. africana, D. mutila, D. seriata, D. scrobiculata, Botryosphaeria dothidea, Lasiodiplodia gonubiensis, L. theobromae* and *Trichoderma paraciridescens* isolates used for the *D. sapinea* species-specific primer design (described in Section 2.4.) was confirmed by sequencing the internal transcribed spacer (ITS) region. ITS-PCR was performed using the fungal-specific PCR primers ITS1-F [52] and ITS4 [53]. PCR reactions were carried out as described by Drenkhan et al. [54]. PCR products were sent for sequencing to the Estonian Biocentre in Tartu. The ITS region of samples was sequenced using the primer ITS5 [53]. The sequences were edited using BioEdit version 7.2.5 [55]. BLAST searches for fungal taxa confirmation were performed in GenBank (NCBI). ITS sequences of the isolates were deposited in GenBank (Suppl. Table S2).

### 2.4. Species-Specific Conventional PCR Primer Design

Primers specific to *D. sapinea* were designed in silico based on mitochondrial small subunit ribosomal DNA (mtSSU rDNA) sequences of *D*. *sapinea* and other related species present in the International Nucleotide Sequence Database Collaboration (INSDC) database. The sequences were downloaded and aligned using MAFFT. Aligned sequences were then scanned for regions conserved in all sequences belonging to *D*. *sapinea*, but that contain mismatches in comparison to sequences from other species of *Diplodia*. Specificity of suitable primer sequences was validated using BLASTn searches against the INSDC nucleotide database and sequences. Primer pairs fully complementary to species other than *D*. *sapinea* were discarded. IDT OligoAnalyzer 3.1 was used to select primers with melting temperatures differing by less than 4 °C, and to check the stability of potential homodimers, heterodimers, and hairpin structures.

All PCR experiments were carried out using the following reaction mixture: 5 μL of PCR mastermix with 10 mM MgCl_2_ (5x HOT FIREPol^®^ Blend Master Mix Ready to Load; Solis Biodyne, Tartu, Estonia); 0.5 μL of both forward and reverse primers (20 μM; 0.4 μM final conc.), 18 μL of PCR grade water, and 1 μL of template DNA. The following thermal cycling program was adopted after optimisation using an annealing temperature gradient: 95 °C for 15 min; 35 cycles at 95 °C for 30 s, 61 °C for 30 s, and 72 °C for 1 min; a final step of 72 °C for 10 min. For primer specificity confirmation, DNA from the pure cultures of *D. sapinea*, *D. africana, D. mutila, D. seriata, D. scrobiculata*, *Botryosphaeria dothidea*, *Lasiodiplodia theobromae*, and *Trichoderma paraviridescens* were used (Suppl. Table S2 and Figure S1), as well as DNA from environmental samples of soil, wood and needles infected by *D. sapinea* (data not shown). In total, we used more than 500 samples from 19 countries covering five continents, including North and South America, Europe, Asia and Oceania. The detection limit of primers was determined using 10-fold serial dilutions of a *D. sapinea* pure culture DNA sample, whose concentration was determined using a Qubit fluorometer (Thermo Fisher Scientific, Waltham, MA, USA).

### 2.5. Haplotype Determination

For multilocus haplotyping, 13 microsatellite markers [35,46] were used with fluorescently labelled forward primers (Table 1). Different fluorescent labels (FAM, ATTO532, ATTO550, ATTO565) allowed fragment analysis to be run in a single panel containing amplicons from all 13 loci. PCR reactions were performed in 20 μL reaction volumes, consisting of 2 μL template DNA, a final concentration of 0.3 μM forward and reverse primer, 4 µL 5× HOT FIREPol Blend Master Mix Ready to Load with 10 mM MgCl_2_ and 13 μL PCR grade water. PCR was done as described by Burgess et al. [46] and Bihon et al. [35] with some modifications (see Table 1).

PCR products for fragment analysis were pooled into a single panel and run on an Applied Biosystems 3130XL genetic analyser along with LIZ 500 size standard (Applied Biosystems). Alleles were scored using GENEMAPPER v5.0 (Applied Biosystems, Carlsbad, CA, USA).

### 2.6. Genetic Diversity and Differentiation of Populations

Individuals with identical alleles at all microsatellite loci were considered clones. Individuals from the same country were considered to represent one subpopulation. Two datasets were generated: one containing all individuals, i.e., the non-clone-corrected (non-cc) dataset; and one containing only one individual of each multilocus haplotype per subpopulation, i.e., the clone-corrected (cc) dataset. The cc dataset was used to calculate the total number of different multilocus haplotypes using GENALEX 6.5 [56]. The non-cc data set was used for calculating the clonal fraction index CF = 1 − [(number of different multilocus haplotypes)/(total number of isolates)] [57]. The clone-corrected (cc) dataset was used to calculate mean haploid genetic diversity (h), total number of alleles, private alleles, mean number of different alleles (Na), and mean unbiased diversity (uh) for each subpopulation using GENALEX 6.5. Allelic richness (A_R_, the number of distinct alleles in a population) and private allelic richness (PA_R,_ the number of alleles unique to a particular population), were calculated with ADZE 1.0 using the rarefaction approach, with subpopulation sizes standardised to the smallest sample size of 6 [58].

An analysis of molecular variance (AMOVA) was used on the cc dataset to test the significance of differentiation between subpopulations. Subpopulation RUS was omitted from AMOVA analyses due to its small sample size which did not meet the requirements of the test. Additionally, an AMOVA was conducted to test differentiation of the subpopulations on the two main hosts, *P. nigra* and *P. sylvestris*. Other host taxa were discarded from the analysis due to their small sample size.

The software STATISTICA was used to test whether mean haploid genetic diversity (h), mean unbiased diversity (uh), allelic richness (A_R_) or private allelic richness (PA_R_) correlate significantly with northern latitudes or eastern longitudes or with disease severity index (Suppl. Text S1).

EDENetworks v2.18 was used for minimum spanning network visualisation on the cc and non-cc dataset [59]. Analyses were carried out on Fst fixation indexes.

### 2.7. Mating Type Determination and Random Mating

To determine the mating type of *D. sapinea* isolates, mating type primers were used to amplify the *MAT* genes. The 20 μL PCR reaction mix consisted of 4 μL of 5× HOT FIREPol Blend Master Mix Ready to Load with 10 mM MgCl_2_, and each mating type primer at a final concentration of 0.5 µM: DipM1f, DipM1r or DipHMGf, DipHMGr [37], 1 μL template DNA and 13 μL PCR grade water. PCR conditions followed Bihon et al. [37], with the adjustment of the initial denaturation step to 95 °C for 12 min.

In order to investigate the possibility of sexual recombination, two tests were carried out on both cc and non-cc datasets for subpopulations represented by at least six isolates. Firstly, an exact binomial test, using two-tailed *p*-values, was used to test whether mating type ratios deviated from a 1:1 ratio. Secondly, the index of association (I_A_) was used to test for haploid linkage disequilibrium of the 13 microsatellite loci in Genalex 6.5 [56].

### 2.8. Isolation by Distance

Mantel tests, conducted in Genalex 6.5, were used to test for isolation by distance on the cc dataset using Nei’s genetic distance [60,61] and geographic distance. Only subpopulations with a sample size of six or higher were included in the analysis. For visualisation of Nei’s genetic distance and geographic distance, principal coordinates analysis (PCoA) was carried out in GENALEX 6.5 using the covariance standardised method on the cc dataset.

### 2.9. Population Clustering

STRUCTURE 2.3.4 [62] was used to determine the most likely number of population clusters (K). Each of 20 independent runs of K = 1−20 were carried out with 100,000 burn-in iterations followed by a run of 500,000. The optimum number of clusters (K) was determined using the ln(Pr(X|K)) method [63,64] in CLUMPAK [65].

## 3. Results

### 3.1. Identification of Diplodia sapinea with Conventional PCR Primers

As a result of in silico screening for inclusivity, specificity, melting temperatures and stability of homodimers, heterodimers and hairpin structures, the following *D. sapinea* specific primer pair was selected: DiSapi-F-5′ CCCTTATATATCAAACTATGCTTTGT 3′ and Diplo-R-5′ TTACATAGAGGATTGCCTTCG 3′. The forward primer is fully complementary only to the sequences of *D. sapinea* (4 bp differ from *D*. *scrobiculata*), whereas the reverse primer is also complementary to *D. scrobiculata*. Together, these primers amplify a 546 bp fragment of *D. sapinea* mtSSU rDNA (Suppl. Figure S1). The detection limit of *D. sapinea* DNA with these primers in DNA extracts of pure cultures was 2.4 pg. The species-specific PCR primers were tested on several isolates of *D. sapinea* from five continents (North America, South America, Europe, Asia and Oceania) (Suppl. Table S2 and Figure S1). In agarose gel electrophoresis, the primers produced a visible band of the expected size only from DNA extracted from *D. sapinea* pure cultures and wood infected by *D. sapinea*. No bands were observed when using DNA from other species or from the 10 analysed soil samples [66].

The new primers Disapi-F and Diplo-R were able to discriminate *D. sapinea* from *D. africana, D. mutila, D. seriata, D. scrobiculata, Botryosphaeria dothidea, Lasiodiplodia gonubiensis, L. theobromae* and *Trichoderma paraciridescens* isolates (Suppl. Table S2 and Figure S1). A total of 425 European isolates were identified as *D. sapinea* using the species-specific primer pairs DiSapi-F/Diplo-R. Isolates which were not *D. sapinea* were excluded from population analyses.

### 3.2. Multilocus Haplotypes

The 425 analysed *D. sapinea* isolates from 15 countries across Europe harboured 76 different alleles in 13 microsatellite loci (Suppl. Table S3). Nine of the 13 microsatellite markers were polymorphic, with locus SS12 harbouring nine alleles, SS5 and SS14 five alleles each, SS9 four alleles, SS7 three alleles, and SS8, SS10, SS13 and SS15 two alleles each. Four markers (SS1, SS2, SS11 and SS16) were found to be monomorphic. 

Fifty-two different multilocus haplotypes (MLH) were found in the 425 European isolates of *D. sapinea* (Table 2). The most frequent haplotype, MLH29, was represented by 185 isolates from 13 subpopulations. The second and third most frequent haplotypes were represented by 37 isolates from nine subpopulations (MLH33) and by 32 isolates from two subpopulations (MLH8) (Table 2). From all 52 haplotypes, 27 were private haplotypes, i.e., found just in a single subpopulation.

Subpopulations GER, GEO, EST and UKR had the highest number of haplotypes (13, 10, 9 and 9, respectively) and GER has the highest number of private haplotypes (7), not represented in other subpopulations (Table 3). In general, *D. sapinea* subpopulations in Europe are characterised by a high clonal fraction index that in 13 out of 15 subpopulations fell in the range 0.45–0.90 (Table 3). Lowest clonal fraction index was found in FIN (0.45) and ITA (0.46) subpopulations, while the highest was recorded in SLO (0.85) and POL (0.88) subpopulations (Table 3).

The highest number of shared haplotypes (5) were found between the EST and GER subpopulations followed by the FIN and POL subpopulations with four shared haplotypes (Table 2).

**Figure 2:**
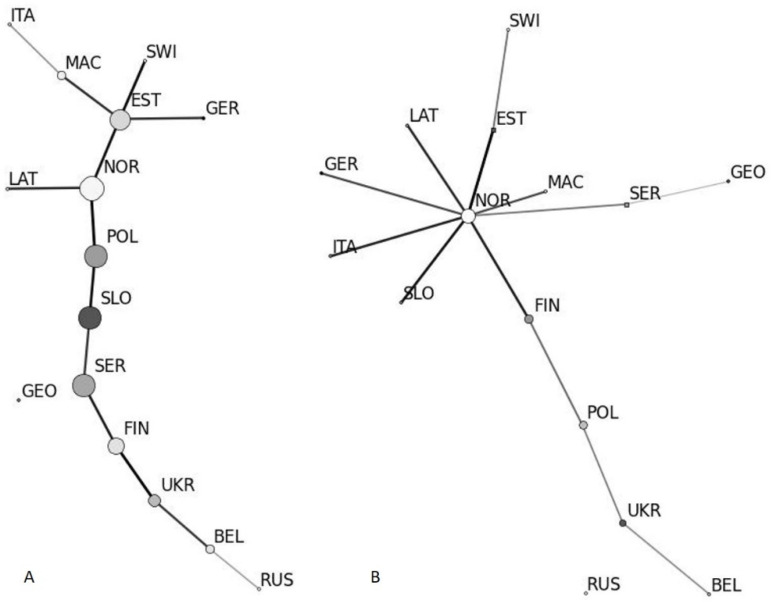
Minimum spanning network of *D. sapinea* subpopulations in Europe and western Asia based on the non-cc dataset (**A**) and cc-dataset (**B**). Darker node colour represents higher haplotype number in subpopulations and node size represents betweenness of subpopulation in the network. Darker edges represent a higher degree of connectivity. Subpopulations are coded as follows: Belarus—BEL, Estonia—EST, Finland—FIN, Georgia—GEO, Germany—GER, Italy—ITA, Latvia—LAT, North Macedonia—MAC, Norway—NOR, Poland—POL, European part of Russia—RUS, Serbia—SER, Slovakia—SLO, Switzerland—SWI and Ukraine—UKR.

In the Estonian subpopulation of first arrivals (several isolates per tree) (EST) of *D. sapinea*, haplotype numbers MLH4, MLH8 and MLH10 were found only in subpopulations EST and GER, while haplotypes MLH29 and MLH33 were found in most examined subpopulations, and haplotype MLH15 was found in two neighbouring countries (Finland and Latvia) to Estonia and in SER (Table 2). In the Estonian subpopulation of the second wave of arrivals (EST), haplotypes MLH2 and MLH16 were found in the EST and UKR subpopulations, MLH9 in EST and GER subpopulations, MLH15 in EST, FIN, LAT and SER subpopulations, MLH40 in EST, GER, ITA, SLO and SWI subpopulations and haplotype number MLH50 in most of the analysed subpopulations (Table 2). Haplotypes MLH1, MLH13, MLH27 and MLH48 were found only in the Estonian (EST) subpopulation. According to minimum spanning network analyses, the betweenness and connectivity between EST an GER population is high on cc and non-cc datasets (Figure 2). GEO subpopulation is related weakly with rest of European subpopulations according to minimum spanning network.

### 3.3. Population Differentiation

**Figure 3:**
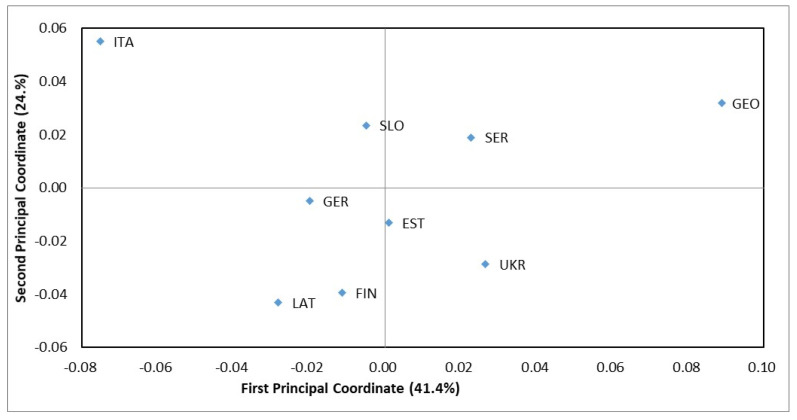
Principal coordinate analysis of Nei’s genetic distance of nine subpopulations of *D. sapinea*. The variation explained by each coordinate is given as a percentage within parentheses. Subpopulation codes: Estonia—EST, Finland—FIN, Georgia—GEO, Germany—GER, Italy—ITA, Latvia—LAT, Serbia—SER, Slovakia—SLO and Ukraine—UKR.

Most European subpopulations of *D. sapinea* did not differ from each other according to the AMOVA of their haplotype variance. Only GEO and GER differed from some other subpopulations (Table 4). According to the AMOVA 97% of the total molecular variance was ascribed to within-population variation and 3% to among-population variation. No significant differentiation (*p* = 0.427) was found between isolates from the two main host species, *P. nigra* and *P. sylvestris*. Calculation of Nei’s genetic distances revealed that subpopulations GEO and ITA clearly differ from other subpopulations in Europe (Table 5, Figure 3). SER subpopulation is distant from EST and LAT.

### 3.4. Isolation by Distance and Clustering Analysis

**Figure 4:**
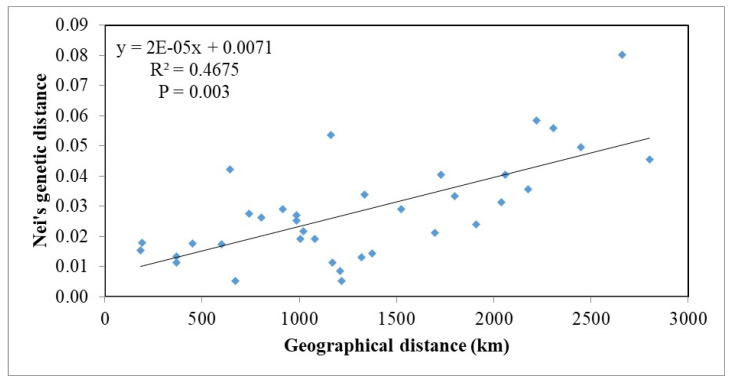
Genetic differentiation of *D. sapinea* subpopulations in Europe and Georgia according to geographical distance. Nei’s genetic distances of isolates representing 15 subpopulations are presented as a function of geographic distance.

The Mantel test on Nei’s genetic and geographical distances revealed strong isolation by distance (*p* = 0.001) when the 15 subpopulations of *D. sapinea* in Europe and Georgia were considered (Figure 4).

**Figure 5:**
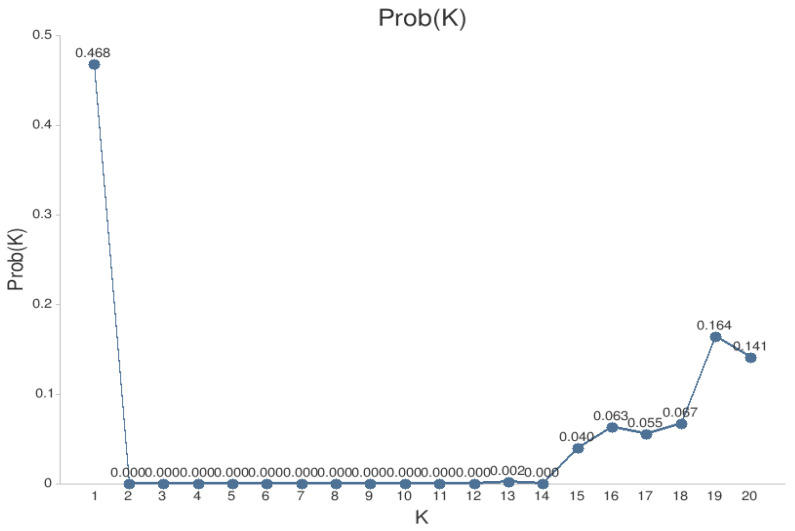
The optimum number of clusters determined using the ln(Pr(X|K)) method [63,64] in CLUMPAK [65].

The Structure results indicated that all of the isolates fell into a single cluster (probability of 0.468) (Figure 5). 

### 3.5. Genetic Diversity and Population Statistics

In the 15 subpopulations analysed with 13 microsatellite markers, 13 (RUS) to 22 (GEO, GER and ITA) alleles were recorded per subpopulation (Table 6). Private alleles were observed in seven subpopulations: one allele in subpopulations BEL, EST, FIN and UKR, two alleles in GER and three alleles in subpopulations GEO and four in ITA. The rest of the subpopulations (LAT, MAC, NOR, POL, RUS, SER, SLO and SWI) did not have private alleles. The highest allelic richness (A_R_) was recorded in subpopulation ITA (1.560) followed by GEO (1.478) and SLO (1.472), but the highest private allelic richness (P_AR_) was found in subpopulation ITA (0.321) followed by GEO (0.152) and GER (0.077) (Table 6). The lowest allelic richness was observed in EST (1.224), while the lowest private allelic richness occurred in SER (0.022). The highest mean number of different alleles (Na) occurred in SLO (1.538), GEO, LAT and ITA (1.462 for each), while lowest values were observed in EST (1.231).

The highest mean unbiased diversity (uh) was found in SLO (0.185), followed by ITA (0.179) and SER (0.174). The lowest mean unbiased diversity was observed in the EST and UKR subpopulations (Table 6). In comparison, high mean haploid genetic diversity (h) was documented in the SLO (0.154), ITA (0.150) and SER (0.143) subpopulations (Table 6), while the lowest values were found in the EST and UKR subpopulations.

No statistically significant correlations between northern latitudes, eastern longitudes or disease severity index and mean haploid genetic diversity (h), mean unbiased diversity (uh), allelic richness (A_R_) or private allelic richness (PA_R_) were found (data not shown).

### 3.6. Mating Type Distribution and Haploid Linkage Disequilibrium

Both mating type idiomorphs (*MAT1-1-1* and *MAT1-2-1*) were represented in 12 out of 15 subpopulations (Table 7). In subpopulation MAC only the *MAT1-2-1* mating type idiomorph was found, while in subpopulations NOR and RUS only the *MAT1-1-1* mating type idiomorph was found (Table 7).

An unequal distribution of mating type idiomorphs was registered in GEO, ITA, MAC, NOR and SLO subpopulations in the non-cc dataset (*p* < 0.05), whereas in the cc dataset both mating type idiomorphs were present in equal proportion (*p* > 0.05) in all subpopulations.

Random mating was not supported by the index of association (I_A_) test in the MAC subpopulation (*p* = 0.045) using the cc dataset, and in the GEO, ITA, MAC, POL, SER and SLO subpopulations using the non-cc dataset (*p* < 0.05). Using both datasets (cc and non-cc) most subpopulations had low linkage disequilibrium, except the MAC subpopulation (I_A_ = 4.168 on non-cc and 6.210 on cc dataset) (Table 7). The significant IA for many subpopulations, together with the balanced ratio of mating types, suggests that sexual reproduction is likely occurring in these subpopulations, albeit at a low level. The high clonal fraction of many subpopulations demonstrates the predominance of asexual reproduction. 

### 3.7. Haplotypic Diversity at Small Spatial Scale

**Figure 6:**
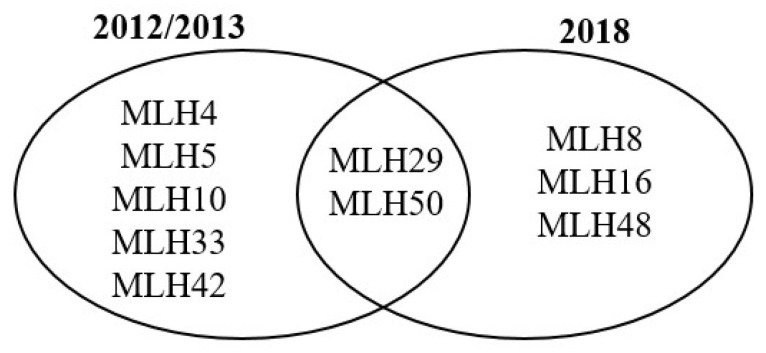
Venn diagram showing *D. sapinea* haplotype distribution from a single *P. nigra* tree in Järvselja nursery, Estonia from two sampling dates.

In 2007 *D. sapinea* was documented for the first time in the Baltic region on cones of a single *P. nigra* tree in Järvselja nursery [25]. Sixteen isolates of *D. sapinea* were obtained from this tree over the course of three years (2012, 2013, 2018) (Table 8). Ten different haplotypes were found from the 16 isolates, giving a clonal fraction of 0.38. The most abundant haplotypes were MLH29 and MLH50, each represented by four isolates. When split by sampling time, 2012/2013 (N = 8) vs. 2018 (N = 8), two haplotypes (MLH29 and MLH50) were found at both sampling times (data not shown). In 2012/2013 seven haplotypes occurred, nos. MLH4, MLH5, MLH10, MLH29, MLH33, MLH42, MLH50 and in 2018 five haplotypes occurred, nos. MLH8, MLH16, MLH29, MLH48, MLH50 (Figure 6).

The first finding of *D. sapinea* on a native pine tree in Estonia was from Vormsi island, from where six *P. sylvestris* trees were sampled in 2012. Ten isolates from six trees consisting of four different haplotypes were obtained, giving a clonal fraction of 0.60. The most abundant haplotypes were no. MHL48 with six representatives followed by no. MHL8 with two representatives (Table 8).

In Muhu island, western Estonia *D. sapinea* has been found since 2008 in a *P. nigra* stand of 0.7 hectares. In 2012 14 isolates of the pathogen were isolated from c 10 trees, which consisted of four different haplotypes, giving a clonal fraction of 0.71. The most abundant haplotypes were nos. MLH29 and MLH45, each occurring six times (Table 8).

In Slovakia 23 different trees from 10 different sites were sampled (Table 8). From each tree two to four isolates were obtained, with each isolate from a different cone. All trees had only one haplotype per tree, with the exception of a single tree in Galanta, from which two haplotypes, no. MHL39 and MHL40, occurred in four isolates (Table 8).

### 3.8. Allele Polymorphism in Different Loci in North America and Europe

To date, from the SSR markers which were used on the isolates from Europe and North America only marker SS6 was found to be monomorphic on both continents (Table 9). Within Europe markers SS1 and SS2 appear monomorphic. Among loci that have been analysed in both continents higher polymorphism is observed in North America than in Europe, while the sample size in North America (N = 67) is roughly 10 times smaller than in Europe (N = 623) (Table 9). In other words, European allele diversity is considerably lower than in North America.

## 4. Discussion

Population analyses, based on 342 isolates of *D. sapinea* from 14 European and 1 western Asian country, revealed that the subpopulations are dominated by one microsatellite haplotype (MLH29), which comprised 45.3% of the isolates (Table 2). Nonetheless, 27 of the 52 recorded multilocus haplotypes were found only once. Only two subpopulations (GEO and ITA) out of the 15 differed significantly from other subpopulations. Significant isolation by distance was found in the European subpopulations (Figure 4), yet they have relatively low Nei’s genetic distance (0.005–0.080) (Table 5) compared with, for example, *Dothistroma septosporum* (0.055–0.857) [67,68]. Due to the clustering analyses grouping all isolates into a single cluster (Figure 5) and a relatively low occurrence of private alleles (Table 6), we conclude that the European *D. sapinea* subpopulation is homogenous and little differentiated, except subpopulations ITA and GEO.

The Italian (ITA) and Georgian (GEO) subpopulations clearly deviated from the others according to Nei’s genetic distance (Figure 3) which may reflect their biogeographic isolation from Central and Northern Europe due to the Alps and Caucasus mountains, respectively. These subpopulations are characterised by having a relatively high number of private haplotypes and alleles (Table 3 and Table 6). Additionally, the ITA and GEO subpopulations were characterised by a high allelic and private allelic richness, mean number of different alleles, mean unbiased diversity and mean haploid genetic diversity compared to other subpopulations.

Based on systematic annual forest disease monitoring (since 2007) on more than 60 permanent sampling sites, *D. sapinea* was distributed randomly around Estonia until 2012 [69]. Since 2013 the pathogen has spread widely by moving from south to north across Estonia [23]. Among the first arrived haplotypes (isolates obtained up to 2012, Table 2) only MLH4, MLH8 and MLH10 were found in subpopulations EST and GER. Haplotypes MLH29 and MLH33 were found in most of the analysed subpopulations in Europe, and haplotype MLH15 was found in neighbouring countries (Finland and Latvia). Additionally, a strong link between Estonian and German subpopulations was demonstrated by minimum spanning network analyses. Therefore, it is likely that the first introductions of *D. sapinea* to Estonia were of German origin or from another, unsampled, population. That the movement of *D. sapinea* infected plant material to Estonia from other countries is possible is demonstrated by the finding of imported seedlings testing positive for the pathogen (R. Drenkhan, unpublished data). For example, these seedlings (*Tsuga canadensis* and *Picea pungens*) were imported from the Netherlands and Germany in November 2015 and displayed no clear symptoms of *D. sapinea* infection, indicating that asymptomatic plants can harbour and facilitate spread of the pathogen.

A previous population study of *D. sapinea* with isolates from Sweden, Italy, Estonia, Spain and Turkey demonstrated the distribution of one haplotype in all investigated countries [24]. Similarly, in the present study, 25 out of 52 haplotypes occurred in at least two different subpopulations (Table 2, Figure 3), across distances of up to 2800 km (GER-GEO) (Table 5). Burgess et al. [47] documented the occurrence of a single haplotype (MS1) in North America, Europe and New Zealand. In the current study, 45.3% of European isolates were represented by haplotype MLH29. According to McDonald and Linde [38] the domination of a few (virulent) genotypes can evolve through a mixed reproduction system, which poses the highest risk to the host. During sexual recombination new genotypes are produced, and the fittest ones are widely and rapidly dispersed by subsequent asexual reproduction. If a haplotype with high fitness is dispersed over a wide area, an epidemic can arise [38]. For a long time, *D. sapinea* was thought to be an asexual fungus [70], but both this study (1:1 distribution of mating types and loci that are not linked) and previous ones demonstrate that *D. sapinea* may be reproducing sexually, at least to some extent [37,45].

*D. sapinea* is native to pine forests of the Northern Hemisphere [47]. In general, plant pathogens are thought to be more diverse in their native area than in areas where they have been recently introduced [71]. Nonetheless, the highest microsatellite diversity was observed in South Africa, although this is due to multiple introductions since 1909 [45,72]. Bihon et al. [45], using the same set of 13 microsatellites as used in this study, found polymorphism in the South African population of *D. sapinea*, while no polymorphism was found among isolates from South America, Europe, Australia, New Zealand and part of Africa [36,42,47,48]. In our study four loci (SS1, SS2, SS11 and SS16) were monomorphic (Table 9; Suppl. Table S3) among 425 isolates. Of the 16 microsatellite loci that have been described for *D. sapinea*, 10 are polymorphic in European subpopulations while 15 are polymorphic in North American populations (Table 9) [42,43,47,48]. In addition, the loci which were analysed in European and North American populations were found to be more diverse in North America, even if the sample size was roughly 10 times smaller than that of Europe (Table 9). These results support the view that *D. sapinea* is more likely indigenous to North America than Europe, while almost nothing is known about the population diversity of the fungus in Asia and thus the exact origin of the pathogen remains unknown.

Somewhat surprisingly, in the Estonian subpopulation a high haplotype diversity was observed on a single tree in Järvselja nursery, southeast Estonia, where 10 haplotypes were found from 16 isolates (Table 8). The *P. nigra* tree was moderately to heavily infected by *D. sapinea*, depending on the year. For comparison, in Slovakia just nine different haplotypes were found among 70 isolates from 23 trees. Only once were two different haplotypes observed on the same tree in Slovakia (Table 8). Of note is that from the single tree in Järvselja two haplotypes (MLH5 and MLH42) were isolated which were not found anywhere else in Estonia and haplotype MLH48 was found only in Estonia (Table 8). Additionally, the first record of *D. sapinea* for Estonia and for all of northern Europe was on this particular *P. nigra* tree [25]. It can be surmised that different haplotypes of *D. sapinea* have been sporadically imported into this area, probably with infected nursery stock. Another possibility is that sexual recombination has occurred on the tree, altering the haplotypic composition of its isolates. Strengthening the hypothesis about sexual proliferation is the occurrence of both mating types on the tree since 2012/2013.

The relatively high genetic diversity observed in newly established subpopulations in the Baltics and Finland may be explained by a long existence of the fungus in endophytic or asymptomatic modes. In the autumn of 2014, a survey was carried out in the northern Baltics including 85 asymptomatic pine needle and bud samples from 14 stands across Estonia and northern Latvia. The samples were analysed with *D. sapinea* species-specific primers and none of the samples were positive for *D. sapinea* (R. Drenkhan, unpublished data). Consequently, the endophytic or asymptomatic existence of *D. sapinea* in Estonia was possible only in restricted or unsampled areas. Terhonen et al. [73] found recently that *D. sapinea* is an endophyte in healthy *P. sylvestris* trees in Finland. Separate introductions of different strains of the fungus, as has happened in South Africa where the highest diversity of the pathogen is documented [45], is the most likely means of increasing genetic diversity of such an invasive pathogen. The distribution of identical haplotypes in remote geographic areas indicates that movement of haplotypes across large distances is possible.

Several molecular assays have been developed for the detection *D. sapinea* in environmental samples [13,14,15,16]. All these previously published methods were tested on isolates originating from 3–6 countries from one or two continents per method [13,14,15,16], while samples from most of Asia and South America were missing. In this study, novel species-specific PCR primers were designed and samples from five continents were used for detection and testing of *D. sapinea* in environmental samples such as imported and planted stock, seed and seedling lots. New species-specific primers provide more rapid and reliable detection of *D. sapinea* compared to conventional or nested conventional PCR primers previously published.

During testing of the new PCR protocol with various isolates of *Diplodia* spp. we identified *D. africana* (MW332343) from a cone collected in California, USA which is to our knowledge the first record of this fungus on *Pinus canariensis* in North America. We also found *D. africana* (MW332342) from a cone of *P. canariensis* collected in the Canary Islands, to our knowledge the first record of *D. africana* in Spain.

## 5. Conclusions

In the European *D. sapinea* population the dominance of a single haplotype as well as high genetic similarity between different regions were found, suggesting high gene-flow between different countries, except Italy and Georgia. The first established subpopulation of *D. sapinea* in northern Europe (Estonia) was likely introduced from central Europe (Germany), due to the occurrence of several common haplotypes. Furthermore, the relatively new subpopulations in northern Europe seem to have a mixed mode of reproduction (both asexual and sexual) and have similar levels of genetic diversity as older subpopulations in central and southern Europe. This situation may suggest that the pathogen population are evolutionary fit and could pose an increasing risk to pine forests, particularly in the face of a changing climate. Despite c 200 years of documented presence in European coniferous forests, *D. sapinea* is less diverse in Europe than in North America, which may suggest that the pathogen is not native to Europe. Further speculations on the origin of *D. sapinea* need population studies to be extended to Asia and Central America.

## Figures and Tables

**Table 1:** Thirteen *D. sapinea* microsatellite markers with fluorescent labels and annealing temperatures. Fluorescent labels were attached to the forward primers. Deviations from the reference labels and annealing temperatures are underlined and highlighted in bold.

Primer Pair	Locus	Fluorescent Label	Annealing Temp (°C)	Reference
TB1 and TB2-2	SS1	**ATTO550**	58	[46]
TB5 and TB6	SS2	**ATTO532**	58	[46]
TB19 and TB20-2	SS5	**FAM**	62	[46]
TB23 and TB24	SS7	**ATTO532**	62	[46]
TB35-2 and TB36	SS8	**ATTO565**	62	[46]
TB37 and TB38	SS9	**ATTO550**	62	[46]
TB41 and TB42	SS10	**FAM**	62	[46]
TB43 and TB44	SS11	**ATTO565**	58	[46]
WB1-a and WB1-b	SS12	**ATTO565**	66	[35]
WB2-a and WB2-b	SS13	FAM	**57**	[35]
WB4-a and WB4-b	SS14	**ATTO550**	**60**	[35]
WB7-a and WB7-b	SS15	**ATTO550**	55	[35]
WB8-a and WB8-b	SS16	**ATTO532**	55	[35]

**Table 2:** Haplotype frequencies of *D. sapinea* in Europe. Occurrence of 52 multilocus haplotypes (MLHs) (based on 13 microsatellite markers) among isolates of *D. sapinea* isolated in 14 European countries and Georgia.

MLH No.	BEL	EST	FIN	GEO	GER	ITA	LAT	MAC	NOR	POL	RUS	SER	SLO	SWI	UKR	Haplotype Frequency	Percent of Total
1	-	(1)	-	-	-	-	-	-	-	-	-	-	-	-	-	(1)	0.0
2	-	1	-	-	-	-	-	-	-	-	-	-	-	-	1	2	0.6
3	-	-	-	-	-	1	-	-	-	-	-	-	-	-	-	1	0.3
4	-	**(1)**	-	-	1	-	-	-	-	-	-	-	-	-	-	1 (1)	0.3
5	-	**(1)**	-	-	-	-	-	-	-	-	-	-	-	-	-	(1)	0.0
6	-	-	-	-	1	-	-	-	-	-	-	-	-	-	-	1	0.3
7	-	-	-	-	1	-	-	-	-	-	-	-	-	-	-	1	0.3
8	-	**1 (7)**	-	-	24	-	-	-	-	-	-	-	-	-	-	25 (7)	7.3
9	-	1	-	-	1	-	-	-	-	-	-	-	-	-	-	2	0.6
10	-	**(1)**	-	-	1	-	-	-	-	-	-	-	-	-	-	1 (1)	0.3
11	-	-	-	-	1	-	-	-	-	-	-	-	-	-	-	1	0.3
12	-	-	-	1	-	-	-	-	-	-	-	-	-	-	-	1	0.3
13	-	(1)	-	-	-	-	-	-	-	-	-	-	-	-	-	(1)	0.3
14	-	-	-	-	-	-	-	1	-	-	-	-	-	-	-	1	0.3
15	-	**(3)**	2	-	-	-	1	-	-	-	-	2	-	-	-	5 (3)	1.5
16	-	(1)	-	-	-	-	-	-	-	-	-	-	-	-	2	2 (1)	0.6
17	-	-	-	2	-	-	-	-	-	-	-	-	-	-	-	2	0.6
18	-	-	-	7	-	-	-	-	-	-	-	-	-	-	-	7	2.0
19	-	-	-	10	-	-	-	-	-	-	-	6	3 (2)	-	-	19 (2)	5.6
20	-	-	-	16	-	-	-	-	-	-	-	-	-	-	-	16	4.7
21	-	-	-	-	-	-	-	-	-	-	-	-	-	-	1	1	0.3
22	-	-	1	-	-	-	3	-	-	1	-	-	-	-	3	8	2.3
23	-	-	-	-	-	-	1	-	-	-	-	-	-	-	-	1	0.3
24	-	-	-	-	-	-	-	-	-	-	-	1	-	-	-	1	0.3
25	-	-	-	4	-	-	-	-	-	-	-	-	-	-	-	4	1.2
26	-	-	-	-	-	-	-	-	-	-	-	-	1 (1)	-	-	1 (1)	0.3
27	-	1	-	-	-	-	-	-	-	-	-	-	-	-	-	1	0.3
28	-	-	-	-	-	-	-	-	-	-	-	-	1 (2)	-	-	1 (2)	0.3
29	3	**5 (14)**	3	3	41	-	7	4	5	27	-	14	36 (16)	1	6	155 (30)	45.3
30	-	-	1	-	-	-	-	-	-	-	-	-	-	-	-	1	0.3
31	-	-	-	-	1	-	-	-	-	-	-	-	-	-	-	1	0.3
32	-	-	-	-	-	-	-	-	-	-	-	5	2	-	-	7	2.0
33	2	**1**	2	1	2	1	-	-	-	1	-	-	8 (12)	-	7	25 (12)	7.3
34	-	-	-	-	-	1	-	2	-	-	-	-	-	-	-	3	0.9
35	-	-	-	-	-	7	-	-	-	-	-	-	-	-	-	7	2.0
36	-	-	-	-	2	-	-	-	-	-	-	-	-	-	-	2	0.6
37	-	-	-	1	-	-	-	-	-	-	-	-	-	-	-	1	0.3
38	-	-	-	-	1	-	-	-	-	-	-	-	-	-	-	1	0.3
39	-	-	-	-	-	-	-	-	-	-	-	-	(1)	-	-	(1)	0.0
40	-	1	-	-	1	1	-	-	-	-	-	-	1 (2)	1	-	5 (2)	1.5
41	-	-	-	-	-	1	-	-	-	-	-	-	-	-	-	1	0.3
42	-	**(1)**	-	-	-	-	-	-	-	-	-	-	-	-	-	(1)	0.0
43	-	-	-	-	-	-	-	-	-	-	-	-	-	-	1	1	0.3
44	-	-	-	-	-	1	-	-	-	-	-	-	-	-	-	1	0.3
45	-	**1 (5)**	-	-	-	-	-	-	-	-	-	-	-	-	-	1 (5)	0.3
46	-	-	-	1	-	-	-	-	-	-	-	-	-	-	-	1	0.3
47	-	-	-	-	-	-	-	-	-	-	1	-	-	-	-	1	0.3
48	-	2 (5)	-	-	-	-	-	-	-	-	-	-	-	-	-	2 (5)	0.6
49	-	-	-	-	-	-	-	-	-	-	-	-	-	-	1	1	0.3
50	4	(4)	2	-	2	-	1	-	-	3	1	1	2 (2)	-	1	17 (6)	5.0
51	1	-	-	-	-	-	-	-	-	-	-	-	-	-	-	1	0.3
52	-	-	-	-	-	-	1	-	-	-	-	-	-	-	-	1	0.3
N	10	14 (45)	11	46	80	13	14	7	5	32	2	29	54 (38)	2	23	342 (83)	100

Haplotypes of first arrivals of *D. sapinea* into Estonia are highlighted in bold and underlined. If several isolates were obtained from single tree or small group of trees then only one randomly chosen haplotype was used and several isolates from same tree were used in small spatial scale analyses and were indicated in brackets. Percentage calculation included haplotypes, which were used in the main study. Subpopulation codes: Belarus—BEL, Estonia—EST, Finland—FIN, Georgia—GEO, Germany—GER, Italy—ITA, Latvia—LAT, North Macedonia—MAC, Norway—NOR, Poland—POL, European part of Russia—RUS, Serbia—SER, Slovakia—SLO, Switzerland—SWI and Ukraine—UKR.

**Table 3:** Number of haplotypes found in the investigated subpopulations of *D. sapinea* and the clonal fraction among isolates of each subpopulation.

Subpopulation Code	N of Sampling Sites	N of Sampled Trees	N of Sampled Insects	N of Isolates	N of Haplotypes cc	N of Private Haplotypes cc	Clonal Fraction
BEL	2	10	-	10	4	1	0.60
EST	11	14	-	14	9	2	0.69
FIN	3	11	-	11	6	1	0.45
GEO	2	46	-	46	10	3	0.78
GER	5	80	-	80	13	7	0.84
ITA	1	13	-	13	7	3	0.46
LAT	8	14	-	14	6	2	0.57
MAC	1	7	-	7	3	1	0.57
NOR	1	-	5	5	1	0	0.80
POL	2	32	-	32	4	0	0.88
RUS	1	2	-	2	2	1	0.00
SER	1	29	-	29	6	1	0.79
SLO	10	54	-	54	8	2	0.85
SWI	1	2	-	2	2	0	0.00
UKR	3	23	-	23	9	2	0.61

Subpopulation codes: Belarus—BEL, Estonia—EST, Finland—FIN, Georgia—GEO, Germany—GER, Italy—ITA, Latvia—LAT, North Macedonia—MAC, Norway—NOR, Poland—POL, European part of Russia—RUS, Serbia—SER, Slovakia—SLO, Switzerland—SWI and Ukraine—UKR.

**Table 4:** Significance of differences in allelic patterns between the investigated subpopulations. AMOVA results are given as *p*-values, with statistically significant *p*-values given in bold and underlined.

Subpopulation Code	BEL	EST	FIN	GEO	GER	ITA	LAT	MAC	POL	RUS	SER	SLO	SWI
EST	0.093												
FIN	0.411	0.435											
GEO	0.150	**0.004**	0.127										
GER	0.138	0.422	0.253	**0.001**									
ITA	0.140	0.470	0.327	**0.013**	0.041								
LAT	0.241	0.454	0.461	0.062	0.249	0.337							
MAC	0.144	0.429	0.318	0.122	0.167	0.439	0.504						
POL	0.234	0.413	0.380	0.140	0.339	0.384	0.481	0.457					
RUS	0.332	0.055	0.287	**0.046**	**0.047**	0.078	0.177	0.096	0.333				
SER	0.388	0.167	0.423	0.390	0.054	0.393	0.445	0.531	0.483	0.322			
SLO	0.430	0.439	0.375	0.163	0.399	0.489	0.438	0.349	0.368	0.117	0.411		
SWI	0.268	0.399	0.355	0.257	0.399	0.332	0.429	0.598	0.539	0.334	0.284	0.438	
UKR	0.378	0.145	0.397	0.152	**0.042**	0.097	0.394	0.409	0.407	0.381	0.392	0.428	0.430

Subpopulation codes: Belarus—BEL, Estonia—EST, Finland—FIN, Georgia—GEO, Germany—GER, Italy—ITA, Latvia—LAT, North Macedonia—MAC, Norway—NOR, Poland—POL, European part of Russia—RUS, Serbia—SER, Slovakia—SLO, Switzerland—SWI and Ukraine—UKR.

**Table 5:** Nei’s genetic and geographical distances for subpopulations with a clone corrected sample size ≥6. Below the diagonal are Nei’s genetic distances and above are geographical distances (km). Genetic differences higher than the arithmetic average value (0.028) are given in bold and underlined.

Subpopulation Code	EST	FIN	GEO	GER	ITA	LAT	SER	SLO	UKR
EST	-	183	2307	1217	1908	190	1524	1209	987
FIN	0.015	-	2446	1318	2057	368	1697	1374	1169
GEO	**0.056**	**0.049**	-	2803	2659	2222	2039	2177	1801
GER	0.005	0.013	**0.045**	-	914	1079	985	672	1004
ITA	0.024	**0.040**	**0.080**	**0.029**	-	1727	643	742	1164
LAT	0.018	0.013	**0.058**	0.019	**0.040**	-	1334	1021	803
SER	**0.029**	0.021	**0.031**	0.025	**0.042**	**0.034**	-	368	601
SLO	0.008	0.014	**0.036**	0.005	0.027	0.022	0.011	-	451
UKR	0.027	0.011	**0.033**	0.019	**0.054**	0.026	0.017	0.018	-

Subpopulation codes: Estonia—EST, Finland—FIN, Georgia—GEO, Germany—GER, Italy—ITA, Latvia—LAT, Serbia—SER, Slovakia—SLO and Ukraine—UKR.

**Table 6:** Diversity statistics of *D. sapinea* for the 15 analysed subpopulations using the clone corrected dataset. For each diversity index the three highest values are given in bold and underlined.

Subpopulation Code	N of Isolates	N of Haplotypes cc	Total No of Alleles	Private Alleles	Allelic Richness Ar (SE) ^1^ cc	Private Allelic Richness Par (SE) ^1^ cc	Mean Number of Different Alleles Na (SE) ^1^ cc	Mean Unbiased Diversity uh (SE) ^1^ cc	Mean Haploid Genetic Diversity h (SE) ^1^ cc	Disease Severity ^2^
BEL	10	4	17	1	NC	NC	NC	NC	NC	2
EST	14	9	17	1	1.224 (0.124)	0.043 (0.043)	1.231 (0.163)	0.072 (0.051)	0.060 (0.042)	1
FIN	11	6	19	1	1.333 (0.154)	0.064 (0.064)	1.385 (0.180)	0.138 (0.063)	0.115 (0.052)	1
GEO	46	10	**22**	**3**	**1.478 (0.257)**	**0.152 (0.103)**	**1.462 (0.243)**	0.149 (0.079)	0.124 (0.066)	2
GER	80	13	**22**	**2**	1.363 (0.159)	**0.077 (0.048)**	1.385 (0.180)	0.138 (0.063)	0.115 (0.052)	2
ITA	13	7	**22**	**4**	**1.560 (0.192)**	**0.321 (0.147)**	**1.462 (0.183)**	**0.179 (0.069)**	**0.150 (0.057)**	2
LAT	14	6	20	0	1.410 (0.159)	0.047 (0.029)	**1.462 (0.183)**	0.169 (0.065)	0.140 (0.054)	1
MAC	7	3	16	0	NC	NC	NC	NC	NC	2
NOR	5	1	14	0	NC	NC	NC	NC	NC	1
POL	32	4	17	0	NC	NC	NC	NC	NC	2
RUS	2	2	13	0	NC	NC	NC	NC	NC	2
SER	29	6	19	0	1.372 (0.171)	0.022 (0.022)	1.385 (0.180)	**0.174 (0.078)**	**0.143 (0.064)**	2
SLO	54	8	19	0	**1.472 (0.187)**	0.032 (0.032)	**1.538 (0.215)**	**0.185 (0.071)**	**0.154 (0.059)**	2
SWI	2	2	15	0	NC	NC	NC	NC	NC	2
UKR	23	9	20	1	1.378 (0.203)	0.043 (0.043)	1.308 (0.175)	0.118 (0.065)	0.098 (0.054)	1

NC—not calculated. SE—standard error. ^1^ Subpopulation sizes are standardised to 6. ^2^ Please see Materials and Methods Section 2.1 and Supplementary Text S1. Subpopulation codes: Belarus—BEL, Estonia—EST, Finland—FIN, Georgia—GEO, Germany—GER, Italy—ITA, Latvia—LAT, North Macedonia—MAC, Norway—NOR, Poland—POL, European part of Russia—RUS, Serbia—SER, Slovakia—SLO, Switzerland—SWI and Ukraine—UKR.

**Table 7:** Mating type distribution and index of association. Significant *p*-values are highlighted in bold and underlined.

Subpopulation Code	*MAT1-1-1* Non-cc	*MAT1-2-1* Non-cc	*p*-Value of Exact Binomial Test Non-cc	Index of Association I_A_ Non-cc	*p*-Value of I_A_ Non-cc	*MAT1-1-1* cc	*MAT1-2-1* cc	*p*-Value of Exact Binomial Test cc	Index of Association I_A_ cc	*p*-Value of I_A_ cc
BEL	5	5	0.623	1.369	0.052	2	2	0.688	1.583	0.339
EST	7	5	0.387	1.384	0.923	4	4	0.637	1.443	1.000
FIN	6	5	0.500	1.640	0.462	3	3	0.656	1.628	0.824
GEO	9	36	**0.000**	1.707	**0.001**	4	6	0.377	1.855	0.169
GER	45	32	0.086	2.081	0.738	10	4	0.090	1.576	0.975
ITA	2	11	**0.011**	2.795	**0.002**	2	5	0.228	2.377	0.346
LAT	5	8	0.291	1.397	0.260	2	4	0.344	1.626	0.721
MAC	0	7	**0.007**	4.168	**0.011**	0	3	0.125	6.210	**0.045**
NOR	5	0	**0.031**	NC	NC	1	0	0.500	NC	NC
POL	20	12	0.108	1.604	**0.005**	3	1	0.313	1.546	0.254
RUS	2	0	0.250	NC	NC	2	0	0.250	NA	NA
SER	14	15	0.500	1.774	**0.001**	4	2	0.344	2.342	0.156
SLO	19	35	**0.020**	1.259	**0.050**	3	6	0.254	1.167	0.890
SWI	1	1	0.750	NC	NC	1	1	0.750	NC	NC
UKR	13	9	0.262	1.715	0.185	7	2	0.090	2.144	0.738

NC—not calculated. Subpopulation codes: Belarus—BEL, Estonia—EST, Finland—FIN, Georgia—GEO, Germany—GER, Italy—ITA, Latvia—LAT, North Macedonia—MAC, Norway—NOR, Poland—POL, European part of Russia—RUS, Serbia—SER, Slovakia—SLO, Switzerland—SWI and Ukraine—UKR.

**Table 8:** Diversity of *D. sapinea* in single trees and small groups of nearby trees.

Country	Location	Sampling Date	Host Species	Substrate	No. of Trees	No. of Isolates	Clonal Fraction	MLH Nos.
Estonia	Järvselja nursery	17.05.2012; 28.06.2013; 16.02.2018	*Pinus nigra*	Cone, shoot	1	16	0.38	4; 5; 8; 10; 16; 29; 29; 29; 29; 33; 42; 48; 50; 50; 50; 50
Estonia	Muhu	25.10.2012	*P. nigra*	Cone	1 stand	14	0.71	8; 15; 29; 29; 29; 29; 29; 29; 45; 45; 45; 45; 45; 45
Estonia	Vormsi	06.09.2012	*P. sylvestris*	Cone, needle	6	10	0.60	2; 8; 8; 29; 48; 48; 48; 48; 48; 48
Slovakia	Arboretum Mlyňany	20.08.2019	*P. sylvestris*	Cone	1	2	0.50	32; 32
Slovakia	Borová Hora	03.09.2019	*P. sylvestris*	Cone	1	2	0.50	29; 29
Slovakia	Borová Hora	03.09.2019	*P. nigra*	Cone	1	3	0.67	29; 29; 29
Slovakia	Galanta	15.08.2019	*P. nigra*	Cone	1	2	0.50	29; 29
Slovakia	Galanta	15.08.2019	*P. nigra*	Cone	1	2	0.50	33; 33
Slovakia	Galanta	15.08.2019	*P. nigra*	Cone	1	3	0.67	33; 33; 33
Slovakia	Galanta	15.08.2019	*P. sylvestris*	Cone	1	3	0.67	50; 50; 50
Slovakia	Galanta	15.08.2019	*P. sylvestris*	Cone	1	3	0.67	29; 29; 29
Slovakia	Galanta	15.08.2019	*P. sylvestris*	Cone	1	3	0.67	33; 33; 33
Slovakia	Galanta	15.08.2019	*P. sylvestris*	Cone	1	4	0.50	39; 40; 40; 40
Slovakia	Hlohovec	13.08.2019	*P. nigra*	Cone	1	3	0.67	28; 28; 28
Slovakia	Kežmarok	04.10.2019	*P. sylvestris*	Cone	1	3	0.67	29; 29; 29
Slovakia	Kežmarok	04.10.2019	*P. nigra*	Cone	1	3	0.67	29; 29; 29
Slovakia	Kežmarok	03.11.2019	*P. sylvestris*	Cone	1	2	0.50	29; 29
Slovakia	Kežmarok	03.11.2019	*P. sylvestris*	Cone	1	3	0.67	29; 29; 29
Slovakia	Nová Lehota	01.10.2019	*P. nigra*	Cone	1	3	0.67	33; 33; 33
Slovakia	Palárikovo	13.08.2019	*P. nigra*	Cone	1	2	0.50	26; 26
Slovakia	Palárikovo	13.08.2019	*P. nigra*	Cone	1	3	0.67	19; 19; 19
Slovakia	Palárikovo	13.08.2019	*P. sylvestris*	Cone	1	3	0.67	33; 33; 33
Slovakia	Stupava	21.03.2019	*P. nigra*	Cone	1	2	0.50	29; 29
Slovakia	Stupava	21.03.2019	*P. nigra*	Cone	1	3	0.67	29; 29; 29
Slovakia	Tematín	01.10.2019	*P. nigra*	Cone	1	2	0.50	33; 33
Slovakia	Vlčany	13.08.2019	*P. nigra*	Cone	1	3	0.67	29; 29; 29

**Table 9:** Number of observed alleles in different microsatellite loci in Northern Hemisphere populations.

Publication Region	N. America [42] *	N. America [47] *	Europe	Europe [48]	Europe [43]	Present Study Europe
N of isolates	16	51	27	85	86	425
Locus						
SS1	5	1	1	1	1	1
SS2	2	2	1	1	-	1
SS3	2	1	1	-	-	-
SS4	2	1	1	-	-	-
SS5	7	4	2	-	1	5
SS6	1	1	1	-	-	-
SS7	5	2	1	1	-	3
SS8	8	1	1	1	-	2
SS9	5	4	1	-	3	4
SS10	2	2	1	1	2	2
SS11	7	7	2	1	1	1
SS12	-	4	1	-	2	9
SS13	-	-	-	3	1	2
SS14	-	-	-	4	3	5
SS15	-	-	-	2	2	2
SS16	-	-	-	1	1	1

* Isolates of North American origin.

## Data Availability

All the ITS sequences are deposited into GenBank (https://www.ncbi.nlm.nih.gov/genbank/, accessed on 16 June 2021). Isolates of *Diplodia sapinea* are stored in the Laboratory of Forest Pathology in the Estonian University of Life Sciences and are deposited in the Fungal Culture Collection, Estonian University of Life Sciences. The collected data are stored on PlutoF Data management and Publishing Platform (https://plutof.ut.ee, accessed on 16 June 2021). All the relevant data are published in this article and its supplementary files.

## Supplementary Materials

The following are available online at https://www.mdpi.com/article/10.3390/jof7080634/s1, Figure S1: Gel picture of amplicons (546 bp) obtained with the species-specific primers DiSapi-F and Diplo-R from various *Diplodia sapinea* isolates (see Suppl. Table S2). The first lane in both rows is a 100–3000 bp ladder. Samples 1–18 represent *D. sapinea* from different regions as follows: 1—Belarus, 2—Brazil, 3—Canada, 4—Estonia, 5—Finland, 6—Georgia, 7—Germany, 8—Italy, 9—North Macedonia, 10—New Zealand, 11—Poland, 12—Russia, Far East, 13—Russia Siberia, 14—Serbia, 15—Singapore, 16—Slovakia, 17—Switzerland, 18—Ukraine. Samples 19–20 represent *D. africana* from Spain, Tenerife and USA, Long Beach. Sample 21 represents *D. mutila* from USA, Long Beach. Sample 22 represents *D. seriata* from Portugal. Samples 23–24 represent *D. scrobiculata* from Canada and Italy. Sample 25 represents *Botryosphaeria dothidea* from Georgia. Samples 26–27 represent *Lasiodiplodia theobromae* from Singapore and *Lasiodiplodia gonubiensis* from Brazil. Sample 28 represents *Trichoderma paraviridescens* from Ukraine. C—negative control. Table S1: *Diplodia sapinea* isolates used in this study. Table S2: List of isolates used for *D. sapinea* specific primers validation. Table S3: Allele frequencies for 13 loci for *D. sapinea*.
