# Supplementary material for: Highly Clonal Structure and Abundance of One Haplotype Characterise the Diplodia sapinea Populations in Europe and Western Asia
Source: J Fungi (Basel). 2021 Aug 4;7(8):634. doi: 10.3390/jof7080634 (PMC8400067; doi:10.3390/jof7080634)
Supplement: Supplementary file 1 [file jof-07-00634-s001.zip › Suppl. Text 1.pdf]

Supplementary Text 1. Disease situation in sampled countries.

Belarus

The first record of *D. sapinea* made in Belarus occurred in 2008. The fungus was found on damaged seedlings of *P. sylvestris* in a forest nursery. A year later massive dying of shoots was observed in a young Scots pine stand [74]. During sampling of cones, needles and shoots of *P. sylvestris* for this study in Dzyarzhynsk District in June 2018 two trees with dead shoot tips, symptoms resembling Diplodia tip blight, were encountered. Cones collected from the ground in the sampling region had only low numbers of *D. sapinea* pycnidia. Similarly, in a nearby seed orchard trees looked healthy overall and some collected cones had low numbers of *D. sapinea* pycnidia.

Estonia

Diplodia sapinea was first observed in Estonia on *P. nigra* in 2007 [25], since 2012 it has been found on *P. sylvestris* [23]. Up to 2013 *D. sapinea* had only a few local distribution areas, mainly on non-native host species. Since 2013, a south to north spread of *D. sapinea* was observed [23]. In 2014-2015, the pathogen was found in forests across Estonia on shoots and cones of *P. sylvestris.* Severe damage was observed on a *P. nigra* stand in Muhu Island in western Estonia, where many shoots in tree crowns were dead. Since 2016, visible symptoms of *D. sapinea* have started to recede in forests and urban greeneries. No serious damage or outbreaks have been observed in annual forest pathology monitoring surveys during 2007 - 2020. In October 2020 only slight shoot damage by *D. sapinea* was observed on *P. nigra* in Muhu Island, where even cones on the forest floor lacked visible pycnidia. Up to 2020 *D. sapinea* has been detected on *P. nigra, P. sylvestris, P. leucodermis, P. mugo* and *Pseudotsuga menziesii*. From 2016-2020, seven records of *D. sapinea* on 1- and 2-year old bare root and container seedlings of *P. sylvestris* in nurseries have been documented.

Finland

No disease outbreaks of *D. sapinea* have so far been reported in Finland though the pathogen has been detected since 2015 on cones in several southern coastal Scots pine forests [26].

Georgia

Diplodia sapinea has been present in Georgia since at least the 1980s [75], but no detailed information about the pathogen can be found. During the sampling for this study in 2015 some *Pinus nigra* and *Pinus sosnovski* trees were observed with typical Diplodia tip blight symptoms in urban greenery of Borjomi, south-central Georgia but the infection rate was low. In a nearby *Pinus sylvestris* var. *hamata* forest, no tip blight symptoms were seen. During sampling in April 2017 severe damage by *D. sapinea* was observed on *P. nigra* in urban greenery and forests in and close to Telavi town, eastern Georgia. Abundant dead branches and shoot tips, as well as some dead trees were observed. Plating of increment drill cores (at stump height) of dead trees resulted in colonies of *D. sapinea.* Fallen cones of *P. nigra* were covered with copious pycnidia of *D. sapinea*. Less damage was observed on *P. sylvestris;* only some dead shoots were recorded, but fallen cones were colonized by pycnidia of the pathogen.

Germany

Diplodia sapinea has been found on both *P. nigra* and *P. sylvestris* in Germany but also on several other coniferous trees (e.g. *Abies alba, A. grandis, Picea abies, and Pseudotsuga menziesii*) and *Fagus sylvatica* [76]. In Germany *D. sapinea* seems to be widely distributed endophytically in Scots pine [10]. Detailed records of disease outbreaks starting in 1990s at the latest are presented by e.g. Langer et al., [19]; Heydeck & Dahms, [77], Petercord & Straßer, [78]; Bußkamp [79], and reports of the disease situation are presented annually in a forest health overview (Übersicht Waldschutz of AFZderWald, [80]). Since 2017 there have been severe outbreaks of Diplodia tip blight in many parts of Germany [80].

Italy

Diplodia sapinea has been present in Italy for more than a century, with the fungus documented in the beginning of the 1900s [81]. Several outbreaks have been recorded in *P. nigra*, *P. halepensis* and *P. pinaster* forests in Italy [82-87]. No remarkable damage by *D. sapinea* was observed in the sampled stand close to Badia Petroia though cones on the forest floor were moderately covered with pycnidia.

Latvia

The first record of *D. sapinea* in Latvia dates to the year 2012 on *Pinus nigra* and *Pinus sylvestris*. Detailed records are reported by Adamson et al. [23]. No outbreaks or severe infection cases have been recorded after this study and no other data are available.

North Macedonia

In the historical park of Skopje, pycnidia of *D. sapinea* were found in 2019 on cones of *P. nigra* on the forest floor. In the City park almost all trees of *P. nigra* showed typical symptoms of Diplodia tip blight and the trees were moderately infected. The pathogen's history in the country is unknown.

Norway

The first record in Norway is from 2002 on dead twigs of *Juniperus communis* in a nursery near the campus of the Norwegian University of Life Sciences at Ås [88]. The isolates used in this paper were isolated from the bark beetle *Pityogenes quadridens* in 2015. No disease outbreaks of *D. sapinea* have so far been reported.

Poland

In September 2017 very slight damage to *P. sylvestris* trees close to Varssavi was recorded. *Diplodia sapinea* was present on cones on the forest floor. No dead branches or shoots were observed and the trees were generally healthy.

Russia, European part

In 2013, *D. sapinea* was first documented on *Pinus sylvestris* in north-west Russia [23]. The samples for this study were collected in 2013 from a naturally regenerated young Scots pine stand in north-western Russia. Only some dead branches or shoots were observed.

Serbia

The first record of *D. sapinea* in Serbia dates back to the 1950s [89]. The sampling for this study was carried out in March 2017 in urban greeneries in Novi Sad. Sampled *P. nigra* and *P. sylvestris* trees were healthy with only mild symptoms of *D. sapinea.* Cones on the ground were covered by pycnidia of *D. sapinea.*

Slovakia

Herbarium specimens of *D. sapinea* deposited in the Plant Pathology Herbarium (NR) of the Institute of Forest Ecology of Slovak Academy of Sciences (IFE SAS), Nitra, Slovakia from the years 1972, 1977, 1978, 1985, 1986 indicate the sporadic occurrence of the pathogen in the

second half of the 20th century. *D. sapinea* was the main pest agent, which played an important role in *P. nigra* dieback from 2000 to 2007. After sanitary cuttings many localities recovered very well [90]. Symptoms of Diplodia tip blight were later recorded on *P. nigra* in some urban greenery localities of Slovakia during the years 2004-2005, 2009, 2014, 2018, in Arboretum Mlňany, and also occasionally in forests. *Diplodia sapinea* was identified predominantly on needles, cones and shoots of *Pinus nigra* which is one of the most widely introduced woody plants in Slovakia. Symptoms of tip blight have also been noted with lower frequency on *P. sylvestris* [91-94]

Switzerland

The presence of *D. sapinea* in Switzerland has been documented since 2002 [95]. In 2018, some dead branches and shoots were observed on sampled Scots pine trees in Sursee, Switzerland. Cones collected from the ground had some pycnidia of *D. sapinea*.

Ukraine

Diplodia sapinea is present in Ukraine [96], but the pathogen history is obscure. Samples were collected in June 2018 from urban greeneries of Lviv and forest areas of Ternopil Oblast. In Lviv, *P. nigra* trees were not noticed to have symptoms on the shoots but cones collected from the ground were covered with *D. sapinea* pycnidia. In Ternopil Oblast *P. sylvestris* and *P. nigra* looked healthy and fallen cones collected had only small numbers of *D. sapinea* pycnidia.

74. Yarmolovich, V.A.; Azovskaya, N.O.; Belomesyatseva, D.B. Diplodiasis—Dangerous disease on young pine trees. *For. Hunt. Econ.* **2010**, *3*, 28–31. (In Russian).
75. Kizikelashvili, O.G. Distribution of some fungus diseases of Pitsunda pine in its habitat. *Mikol. Fitopatol.* **1984**, *18*, 330–333.
76. Langer, G.J.; Bußkamp, J. Botryosphaeriaceae Diseases: Emerging Threats to European Beech in Germany in the Context of Global Warming and Vitality Loss of Beech. *For. Pathol.* **2021**, submitted.
77. Heydeck, P.; Dahms, C. Trieberkrankungen an Waldbäumen im Brennpunkt der forstlichen Phytopathologie. *Eberswalder Forstl Schr.* **2012**, *49*, 47–55.
78. Petercord, R.; Straßer, L. Mit der Trockenheit kommt der Pilz. *LWF Aktuell* **2017**, *112*, 9–11.
79. Bußkamp, J. Schadenserhebung, Kartierung und Charakterisierung des "Diplodia-Triebsterbens" der Kiefer, insbesondere des endophytischen Vorkommens in den klimasensiblen Räumen und Identifikation von den in Kiefer (*Pinus sylvestris*) vorkommenden Endophyten. Universität Kassel, Germany, 2018.
80. Anonymus. Übersicht Waldschutz 2019/2020. AFZ-Der Wald, (11/2020), 2020, 12–15.
81. Petri, L. Disseccamenti dei rametti di Pseudotsuga douglasii Carr. prodotto da una varietà di Sphaeropsis ellisii. *Sacc. Ann. Mycol.* **1913**, *11*, 278–280.
82. Capretti, C. *Diplodia pinea* (Desm.) Kickx agente del disseccamento di varie specie del genere *Pinus* e di altre conifere. *Acad. Ital. Sci. For.* **1956**, *5*, 171–202.
83. Moriondo, F. Le malattie infettive dei rimboschimenti. *Ann. Dell'Accad. Sci. For.* **1976**, *12*, 181–196.
84. Vagniluca, S.; Goggioli, V.; Capretti, P. Cankers and shoot blights of *Pinus pinea* in Italy. In *Proceedings of the Joint Meeting of the IUFRO Working Parties S2.06.02 and S2.06.04 Shoot and Foliage Diseases in Forest Trees. Vallombrosa, Firenze, Italy, 6–11 June 1994*; Capretti, P., Heiniger, U., Stephan, R., Eds.; University of Florence: Florence, Italy, 1995; pp. 284–286. ISBN 88-900074-0-0.
85. Danti, R.; Capretti, P. Shoot blight of *Pinus halepensis* Mill. in the Italian peninsula. Foliage, shoot and stem diseases. In Proceedings of the IUFRO WP 7.02.02 Meeting, Quebec City, Canada, 25–31 May 1997, Information Report Laurentian Forestry Centre, Quebec Region, Canadian Forest Service: LAU-X-122, pp. 103–107.
86. Maresi, G.; Ambrosi, P.; Battisti, A.; Capretti, P.; Danti, R.; Feci, E.; Minerbi, S.; Tegli, S. Pine dieback by *Sphaeropsis sapinea* in Northern and Central Italy. In Forest research Institute Res. Papers. In Proceedings of the IUFRO Working Party 7.02.02 Shoot and foliage Diseases, Hyytiälä, Finland, 17-22 June 2001. 2002; pp. 60–67.
87. Wolynski, A.; Maresi, G.; Ambrosi, P.; Luchi, N.; Capretti, P. *Sphaeropsis sapinea* rimboschimenti di pino nero in Trentino. *Sherwood* **2004**, *102*, 13–17.
88. Talgø, V.; Stensvand, A. *Sphaeropsis sapinea. Grønn Kunnskap e* **2002**, *7*, 2.
89. Karadžić, D. Uticaj patogene mikoflore na propadanje i susenje stabala u kulturama Pinus vrsta. *Šumarstvo* **1987**, *5*, 89–106.
90. Kunca, A. Pine dieback of Austrian pine caused by *Sphaeropsis sapinea* [Hynutie porastov borovice čiernej spôsobované hubou *Sphaeropsis sapinea*]. In *Aktuálne Problémy v Ochrane Lesa 2004*, Varínsky, J. Ed.; Lesnícky Výskumný Ústav: Zvolen, Slovakia, 2004; pp. 170–173.
91. Juhásová, G.; Adamčíková, K.; Kobza, M. Sphaeropsis tip blight disease of Austrian pine in urban greenery. *Hortic. Sci.* **2011**, *33*, 11–15, doi:10.17221/3734-hortsci.
92. Ivanová, H.; Bernadovičová, S. Species diversity of microscopic fungi on Austrian pines growing in urban greenery of Nitra town. *Folia Oecol.* **2010**, *37*, 168–179.
93. Pastirčáková, K.; Ivanová, H.; Pastirčák, M. Species diversity of fungi on pines (*Pinus* spp.) in urban and extra-urban vegetation. In *Dendrological Days in Arboretum Mlyňany SAS 2014: Environment and Trees Vigor*, Barta, M., Ferus, P., Eds.; Mlyňany Arborétum: Vieska nad Žitavou, Slovakia, 2014; pp. 150–157.
94. Ivanová, H. Occurrence of fungi *Sphaeropsis sapinea* and *Sordaria macrospora* on Austrian pine in Mlyňany Arboretum. In *Dendrological Days in Arboretum Mlyňany SAS 2018: Trees and Environment*; Filová, A., Ferus, P., Eds.; Arborétum Mlyňany, Vieska nad Žitavou, Slovakia:, 2018; pp. 80–87.
95. Engesser, R. Red pines in the valleys of Reuss and Limmat. (Rote Föhren im Reuss- und Limmattal.). *Inf. Forsch. Wald.* **2002**, *12*, 5–6.
96. Davydenko, K.V. Sphaeropsis shoot blight in pine plantations under stress conditions. *Вісник Харківського Національного аграрного Університету ім. ВВ Докучаєва. Серія: Фітопатологія та Ентомологія* **2018**, *1–2*, 29–36.
