# Supplementary figures and images for: Highly Clonal Structure and Abundance of One Haplotype Characterise the Diplodia sapinea Populations in Europe and Western Asia
Source: J Fungi (Basel). 2021 Aug 4;7(8):634. doi: 10.3390/jof7080634 (PMC8400067; doi:10.3390/jof7080634)

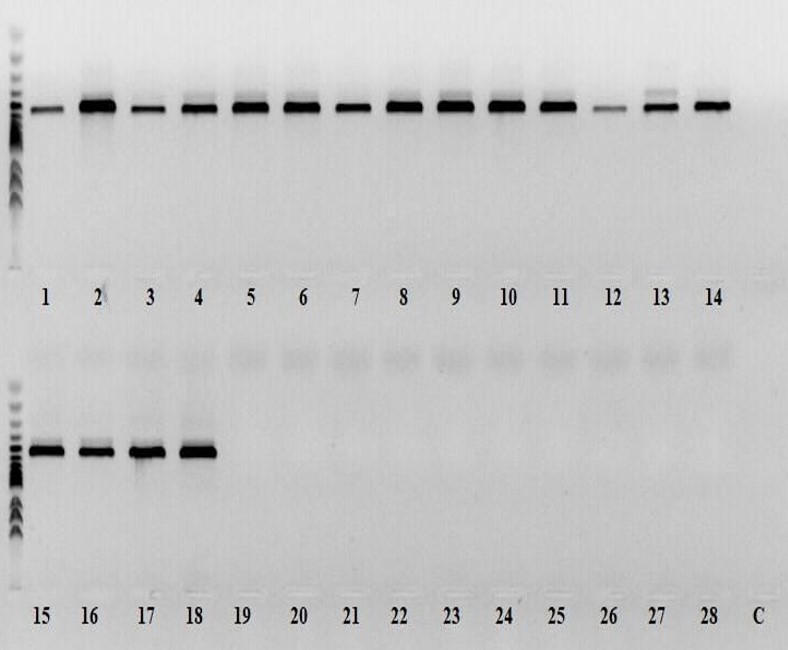

Supplement: Supplementary file 1 [file jof-07-00634-s001.zip › Suppl. Fig. 1.jpg]
